# Sleep Duration, Bedtime, and Myopia Progression in a 4-Year Follow-up of Chinese Children: The Anyang Childhood Eye Study

**DOI:** 10.1167/iovs.61.3.37

**Published:** 2020-03-20

**Authors:** Shi-Fei Wei, Shi-Ming Li, Luoru Liu, He Li, Meng-Tian Kang, Yun-Yun Sun, Yi-Peng Wang, Xiao-Yuan Yang, Ningli Wang

**Affiliations:** 1 Beijing Institute of Ophthalmology, Beijing Tongren Eye Center, Beijing Tongren Hospital, Capital Medical University; Beijing Ophthalmology & Visual Sciences Key Laboratory, Beijing, China; 2 Anyang Eye Hospital, Henan Province, China; 3 Department of Ophthalmology, Zhengzhou Second Hospital, Henan Province, China

**Keywords:** myopia progression, axial elongation, children, sleep duration, bedtime

## Abstract

**Purpose:**

To investigate the relationship between sleep duration and bedtime with myopia progression and axial elongation during a 4-year follow-up in primary school children.

**Methods:**

This study included 1887 children (aged 7.09 ± 0.41 years) who had cycloplegic refractions data at baseline and a fourth visit and 2209 children (aged 7.10 ± 0.41 years) for axial length. All children underwent comprehensive ophthalmologic examinations, including cycloplegic refraction and ocular biometry, and standardized questionnaires, including average night-time sleep duration (h/d) and bedtime (time to bed). Myopia was defined as spherical equivalent < –0.5 diopters.

**Results:**

At the last follow-up, the mean myopia progression and axial elongation for all children were –1.89 ± 1.28 diopters and 1.22 ± 0.57 mm. After stratifying the sleep duration into tertile groups, myopia progression and axial elongation were slower in children with highest sleep duration tertile (*P* = 0.04 and P =0.014) in girls but not in boys, compared with the middle sleep duration tertile. However, after adjusting for potential confounders, no significant association was found for sleep duration with myopia progression and axial elongation for the children (*P* = 0.255 and *P* = 0.068), and the association with axial elongation was only of borderline significance in girls (*P* = 0.045). The bedtime was not associated with myopia progression and axial elongation in the regression analyses (*P* = 0.538; *P* = 0.801).

**Conclusions:**

These results show that there was no significant association between sleep duration and bedtime with myopia progression and axial elongation among children. The findings in girls might be related to the earlier onset of puberty.

The prevalence of myopia has been increasingly reaching an epidemic rate in some areas of East and Southeast Asia in recent decades,^1^ and it has become the major cause of correctable visual impairment globally.^2^^–^^4^ Visual impairment owing to myopia places a heavy cost burden, including correction of visual acuity via glasses, contact lenses, and refractive surgery.^5^ In addition, high or pathologic myopia can result in severe loss of vision and even blindness.^6^^–^^8^ An important measure to enable the effective prevention of myopia is understanding the modifiable risk factors from a public health perspective. Numerous studies have shown that many factors, such as educational pressure, parental myopia, increased near work, and less time spent on outdoor activities seem to contribute to the pathogenesis of myopia.^9^^–^^12^ Considering the high prevalence of myopia, there is an increasing need to explore other environmental factors that may affect the onset and progression of myopia.

Many studies have indicated that children in China sleep less at night than their peers in the United States and elsewhere.^13^^,^^14^ Accordingly, a hypothesis that a lack of sleep may be associated with myopia has been proposed. Recently, some epidemiologic studies have investigated the relationship between sleep duration and myopia.^15^^–^^20^ For example, the Korean National Health and Nutrition Examination Survey found a significant relationship between shorter sleep duration and myopia using population-based data from Korean adolescents aged 12 to 19 years.^15^ Another study in Japanese children found that poor sleep quality was significantly correlated with high myopia.^16^ However, a study on Chinese children investigated the relationship between total (night + midday) sleep and myopia, showing that they were not significantly associated.^17^ Surprisingly, in a community-based study of 5613 Chinese residents aged 60 years and older, Xu et al.^20^ found that people who had a longer night-time sleep duration would have a higher risk of myopia. We hypothesized that this is due to the older adult population in this study. It is possible that there is some other underlying mode for sleep duration affecting myopia in older adults, and there is a need for further related research about the precise mechanisms.

One study in Japanese children explored the relationship between bedtime and myopic refractive error.^16^ The study found that bedtime was significantly associated with myopic refractive error in the regression analyses.

At present, the relationship between sleep duration and myopia remains controversial and has not been extensively evaluated. Furthermore, there is a lack of cohort studies investigating the effects of sleep duration on myopia development and progression, and no study has investigated the relationship between sleep duration and axial length (AL). Furthermore, the relationship between bedtime and myopia has only been investigated in Japanese children.^16^

To address the need for exploring the relationship between sleep duration and bedtime with myopia progression and axial elongation, we carried out this study to investigate the association in a cohort study design over a 4-year follow-up period.

## Methods

### Study Population

The Anyang Childhood Eye Study is a school-based cohort study aiming to observe the prevalence, incidence and risk factors for myopia among Chinese children in urban areas of Anyang city, Henan Province, central China. Details of the methodology have been provided elsewhere.^21^ In brief, 2893 grade 1 students from 11 primary schools were examined from February to May 2012 with a response rate of 93.0% with follow-up once a year. Informed written consent was obtained from at least one parent, as well as verbal assent from each child. The study adhered to the tenets of the Declaration of Helsinki. Ethics committee approval was obtained from the Institutional Review Board of Beijing Tongren Hospital, Capital Medical University.

### Procedures

During the examination of each child, cycloplegia was performed after instilling 1 drop of topical anesthetic agent (Alcaine; Alcon, Fort Worth, TX), followed by two drops of 1% cyclopentolate (Alcon) and one drop of tropicamide (Mydrin P; Santen, Osaka, Japan) at a 5-minute interval. Thirty minutes after the last drop, if the pupillary light reflex was still present or the pupil size was less than 6.0 mm, a third drop of cyclopentolate was administered. Refraction was measured by an autorefractor (HRK7000 A; Huvitz, Gunpo, South Korea) and the average of three measurements was used for analysis. AL was measured using the Lenstar LS900 (Haag-Streit, Koeniz, Switzerland).

An interviewer-administered questionnaire answered by parents was used to collect information on the number of myopic parents, time outdoors, and near work activities (hours per day) of their children after school hours.^11^^,^^21^ Hours spent outdoors and near work activities during the school day were assumed to be approximately constant for the same education system, and so were not individually calculated. Sleep duration (hours per day) and bedtime (hour) were also estimated by this questionnaire. In the present study, we only included night-time sleep duration. Parents were asked: “On weekdays and weekends, when do your children sleep and get up?” The average sleep duration per day was calculated as time spent during weekdays × 5/7 + time spent during weekends × 2/7. To investigate the relationship between sleep duration, bedtime and the progression of myopia, baseline measures for sleep duration, bedtime, time outdoors, and near work were used.^11^^,^^22^

### Definitions

The spherical equivalent (SE) was calculated as the dioptric powers of the sphere and half of the cylinder (sphere + 0.5 × cylinder). Myopia was defined as SE of less than –0.5 diopter (D), hyperopia as greater than +0.5 D, and emmetropia as an SE between –0.5 and +0.5 D. Myopia progression was calculated as the change in the cycloplegic SE between the measurements acquired at baseline and the readings taken at the final follow-up examination. The total axial elongation was defined as the AL at the final follow-up examination minus the AL at baseline. Children were classified to have incident myopia if they were not myopic at baseline and had become myopic at the end of follow-up. The cumulative incidence of myopia was defined as the proportion of subjects who were not myopic at baseline and who subsequently developed myopia during the follow-up period. Time levels were divided into low, middle, and high using population tertiles of the average sleep duration (≤9.56 h/d; 9.57–10.00 h/d; ≥10.01 h/d). Time levels were divided into early, middle, and late levels using population tertiles of the bedtime (9:00 PM or earlier; 9:01–9:59 PM; 10:00 PM or later).

### Statistical Analysis

For all analyses, SPSS version 20.0 (SPSS, Chicago, IL) was used. Because the SE and AL for the right and left eyes were highly correlated (SE, 0.85; AL, 0.82), analyses were performed on the right eye only. Continuous variables were presented as mean ± standard deviation, and categorical variables were presented as percentages. An independent *t*-test and chi-square tests were used to compare the normally continuous data and the categorized data, respectively. We examined the differences in the mean values of myopia progression and axial elongation by an ANOVA with a post hoc Scheffé test. Multivariate linear regression models using a stepwise backward method were constructed. Each value represents the result of a separate regression model, with the individual myopia progression and axial elongation as the dependent variables and sleep duration as the independent variable and adjusting for other covariates, including age at baseline, sex, parental myopia, time outdoors, near work, height, and weight. The multivariate logistic regression analysis using a stepwise backward method was used to explore sleep duration associated with incident myopia. Odds ratios and 95% confidence intervals were presented. A two-sided *P* value of less than 0.05 was considered statistically significant.

## Results

In 2012, the study included 2893 grade 1 students at baseline with a mean age of 7.09 ± 0.41 years (range, 5.67–9.27 years). Of the 2893 grade 1 students, a total of 2328 children (80.5%) had the data of sleep duration and at least one data of cycloplegic refractions or AL at the fourth follow-up examination in 2017. Among the 2328 subjects, 1887 had cycloplegic refractions data at baseline and fourth visits and 2209 for AL. Mean baseline sleep duration of the children was 9.83 ± 0.58 h/d and 57.9% of the children were boys. Table 1 shows the baseline characteristics of the 2328 children. There were no significant differences between boys and girls for SE, sleep duration, number of myopic parents, time outdoors, and near work. However, boys were older, taller, heavier, and have longer AL than girls (Table 1).

**Table 1.** Baseline Characteristics of the 2328 Children

Variable	All	Boys	Girls	*P* Value
Age (years)	7.09 ± 0.41	7.12 ± 0.42	7.06 ± 0.39	<0.01
SE (D)	0.98 ± 0.95	0.95 ± 0.93	1.02 ± 0.98	0.13
AL (mm)	22.71 ± 0.75	22.94 ± 0.69	22.38 ± 0.69	<0.01
Sleep duration, h/d	9.83 ± 0.58	9.84 ± 0.54	9.81 ± 0.64	0.19
Bedtime, h	21.09 ± 0.51	21.08 ± 0.51	21.11 ± 0.50	0.15
Height (cm)	123.60 ± 5.50	124.20 ± 5.49	122.75 ± 5.40	<0.01
Weight (kg)	24.70 ± 4.68	25.29 ± 4.74	23.89 ± 4.46	<0.01
Parental myopia, n (%)				0.57
None	1626 (69.8%)	953 (70.9%)	673 (69.4%)	
One	546 (23.4%)	314 (23.4%)	232 (23.9%)	
Both	142 (6.1%)	77 (5.7%)	65 (6.7%)	
Time outdoors, h/d	1.03 ± 0.79	1.02 ± 0.82	1.04 ± 0.74	0.61
Near work, h/d	1.79 ± 0.92	1.78 ± 0.92	1.80 ± 0.93	0.71

The mean baseline SE was 0.98 ± 0.95 D for 1887 children with cycloplegic refraction and AL was 22.71 ± 0.75 mm for 2209 children. The mean SE of low tertile, middle tertile, and high tertile of average sleep duration was 1.00 ± 1.02 D, 1.00 ± 0.87 D, and 0.94 ± 0.98 D (ANOVA; *P* = 0.43), and the mean AL was 22.72 ± 0.74 mm, 22.70 ± 0.74 mm, and 22.70 ± 0.76 mm (ANOVA; *P* = 0.90), respectively. When the bedtime was divided into early, middle, and late levels, the mean SE was 0.96 ± 0.94 D, 1.00 ± 0.96 D, and 0.92 ± 1.03 D (ANOVA; *P* = 0.45), and the mean AL was 22.70 ± 0.75 mm, 22.71 ± 0.74 mm, and 22.71 ± 0.78 mm (ANOVA; *P* = 0.93), respectively. There were no significant differences between baseline SE and AL with sleep duration and bedtime.

At the last follow-up, the respective mean myopia progression values for all children, boys, and girls were –1.89 ± 1.28 D, –1.71 ± 1.25 D, and –2.10 ± 1.30 D; boys demonstrated a significantly smaller myopic progression than girls (*P* < 0.001). The respective mean axial elongation values for all children, boys, and girls were 1.22 ± 0.57 mm, 1.16 ± 0.56 mm, and 1.29 ± 0.58 mm, respectively; boys also demonstrated a significantly smaller axial elongation than girls (*P* < 0.001). The myopia progression and axial elongation in four years according to sleep duration are shown in Table 2. Sleep duration was not significantly associated with myopia progression and axial elongation for all children (ANOVA; *P* = 0.70; *P* = 0.114). When the analysis was split by sex, there was a significantly decreasing myopia progression and axial elongation with increasing sleep duration in girls (ANOVA, *P* = 0.013; *P* = 0.002) but not in boys (ANOVA, *P* = 0.24; *P* = 0.74). Compared with children in the middle sleep duration tertile, girls in the highest sleep duration tertile had myopia progression of 0.31 D less (ANOVA, *P* = 0.040, post hoc Scheffé test) and axial elongation 0.15 mm shorter (ANOVA, *P* = 0.014, post hoc Scheffé test). However, no significant difference between the highest sleep duration tertiles and low sleep duration tertiles was found in myopia progression and axial elongation (*P* = 0.07; *P* = 0.11). In addition, there was no significant difference between bedtime levels with myopia progression and axial elongation (ANOVA, *P* = 0.66; *P* = 0.88; Table 3).

**Table 2.** The Myopia Progression and Axial Elongation in 4 Years Stratified by Sleep Duration and Sex

Sleep Duration (h/d)	Number	SE (D)	*P* Value^*^	AL (mm)	*P* Value^*^
All			0.70		0.11
≤9.56	748	–1.88 ± 1.30		1.22 ± 0.55	
9.57–10.00	846	–1.92 ± 1.27		1.24 ± 0.58	
≥10.01	734	–1.86 ± 1.29		1.18 ± 0.58	
Boys			0.24		0.74
≤9.56	453	–1.65 ± 1.25		1.14 ± 0.54	
9.57–10.00	465	–1.68 ± 1.14		1.17 ± 0.53	
≥10.01	430	–1.81 ± 1.35		1.17 ± 0.60	
Girls			0.013		0.002
≤9.56	321	–2.15 ± 1.30		1.33 ± 0.55	
9.57–10.00	355	–2.22 ± 1.35		1.34 ± 0.63	
≥10.01	304	–1.91 ± 1.22		1.19 ± 0.56	

* *P* values from ANOVA.

**Table 3.** The Myopia Progression and Axial Elongation in 4 Years Stratified by Bedtime and Sex

Bedtime	SE (D)	*P* Value^*^	AL (mm)	*P* Value^*^
All		0.66		0.88
Early level	–1.90 ± 1.27		1.21 ± 0.57	
Middle level	–1.89 ± 1.29		1.22 ± 0.57	
Late level	–1.80 ± 1.29		1.21 ± 0.58	
Boys		0.71		0.56
Early level	–1.69 ± 1.24		1.14 ± 0.55	
Middle level	–1.74 ± 1.25		1.18 ± 0.56	
Late level	–1.65 ± 1.22		1.16 ± 0.57	
Girls		0.41		0.71
Early level	–2.16 ± 1.26		1.31 ± 0.59	
Middle level	–2.08 ± 1.31		1.28 ± 0.58	
Late level	–1.96 ± 1.36		1.27 ± 0.57	

* *P* values from ANOVA.

Table 4 shows the regression models for sleep duration. After adjusting for potential confounders, no significant association was found for sleep duration with myopia progression and axial elongation (*P* = 0.26 and *P* = 0.07). When the analysis was split by sex, the association between sleep duration and axial elongation in girls is only of borderline significance (*P* = 0.045). The axial elongation significantly decreased by 0.086 mm per 1 hour increase in sleep duration in girls. The magnitude of effects was clinically small. No significant association was found between bedtime and myopia progression and axial elongation in the regression analysis (Table 5).

**Table 4.** Linear Regression Models of Myopia Progression and Axial Elongation by Sleep Duration and Sex

Variable	Unadjusted Model	*P* Value	Model 1	*P* Value	Model 2	*P* Value	R2 Final Models^*^
All							
SE (D)	0.041 (–0.064 to 0.146)	0.45	0.062 (–0.062 to 0.186)	0.33	0.076 (–0.055 to 0.206)	0.26	0.029
AL (mm)	–0.041 (–0.084 to 0.003)	0.07	–0.044 (–0.095 to 0.007)	0.09	–0.050 (–0.103 to 0.004)	0.07	0.038
Boys							
SE (D)	–0.069 (–0.209 to 0.071)	0.33	–0.006 (–0.170 to 0.157)	0.94	0.022 (–0.148 to 0.193)	0.80	0.039
AL (mm)	–0.002 (–0.059 to 0.054)	0.93	–0.013 (–0.078 to 0.052)	0.69	–0.021 (–0.089 to 0.048)	0.55	0.037
Girls							
SE (D)	0.150 (–0.006 to 0.305)	0.06	0.125 (–0.061 to 0.311)	0.19	0.115 (–0.084 to 0.314)	0.26	0.015
AL (mm)	–0.086 (–0.154 to –0.018)	**0.013**	–0.084 (–0.166 to –0.002)	**0.043**	–0.086 (–0.170 to –0.002)	**0.045**	0.036

Data in parentheses represent the 95% CI. Bold values indicate statistical significance (*P* < 0.05).

Model 1 is adjusted for age, gender, and parental myopia.

Model 2 is adjusted for age, gender, parental myopia, time outdoors, near work, height, and weight.

* Two of final regression models.

**Table 5.** Linear Regression Models of Myopia Progression and Axial Elongation by Bedtime and Sex

Variable	Unadjusted Model	*P* Value	Model 1	*P* Value	Model 2	*P* Value	R2 Final Models^*^
All							
SE (D)	0.029 (–0.086 to 0.144)	0.62	0.004 (–0.130 to 0.138)	0.95	–0.014 (–0.153 to 0.126)	0.54	0.033
AL (mm)	0.014 (–0.033 to 0.061)	0.56	0.007 (–0.048 to 0.061)	0.81	0.007 (–0.049 to 0.064)	0.80	0.040
Boys							
SE (D)	0.021 (–0.128 to 0.170)	0.78	–0.049 (–0.222 to 0.124)	0.58	–0.074 (–0.256 to 0.109)	0.43	0.049
AL (mm)	0.026 (–0.035 to 0.086)	0.41	0.026 (–0.043 to 0.094)	0.46	0.023 (–0.050 to 0.096)	0.54	0.045
Girls							
SE (D)	0.071 (–0.105 to 0.248)	0.43	0.110 (–0.095 to 0.315)	0.29	0.100 (–0.111 to 0.310)	0.35	0.026
AL (mm)	–0.012 (–0.087 to 0.064)	0.76	–0.030 (–0.118 to 0.057)	0.50	–0.022 (–0.111 to 0.067)	0.62	0.041

Data in parentheses represent the 95% confidence interval. Bold values indicate statistical significance (*P* < 0.05).

Model 1 is adjusted for age, gender, and parental myopia.

Model 2 is adjusted for age, gender, parental myopia, time outdoors, near work, height, and weight.

* R2 of final regression models.

After 4 years, the cumulative incidence of myopia was 45.9% for all children. A higher percentage of girls had progression of myopia than boys (50.1% vs. 42.6%; *P* = 0.001). Table 6 presents the proportion of children with incident myopia according to baseline sleep duration. We found that girls in the first tertile of sleep duration had a greater incidence of myopia (53.5%) than girls in the third tertile (44.0%). However, incidence of myopia was not associated with sleep duration in the univariate logistic regression analyses. Table 7 presents the proportion of children with incident myopia according to baseline bedtime. The results show that incidence of myopia was not associated with bedtime in the univariate logistic regression analyses (*P* = 0.68).

**Table 6.** Association (Univariate Analysis) between Incidence of Myopia and Sleep Duration

Sleep Duration (h/d)	Incident Myopia, % (n)	Odds Ratio (95% CI)	*P* Value
All			
≤9.56	46.6% (264)	Reference	
9.57–10.00	46.8% (304)	1.012 (0.769–1.333)	0.93
≥10.01	44.3% (259)	0.944 (0.711–1.253)	0.69
Boys			
≤9.56	40.8% (127)	Reference	
9.57–10.00	42.2% (154)	0.848 (0.579–1.242)	0.40
≥10.01	44.7% (142)	1.082 (0.734–1.595)	0.69
Girls			
≤9.56	53.5% (137)	Reference	
9.57–10.00	52.6% (150)	1.240 (0.827– 1.858)	0.30
≥10.01	44.0% (117)	0.790 (0.520– 1.201)	0.27

CI = confidence interval.

**Table 7.** Association (Univariate Analysis) between Incidence of Myopia and Bedtime

Bedtime	Incident Myopia, % (n)	Odds Ratio (95% CI)	*P* Value
All			
Early level	45.9% (302)	Reference	
Middle level	46.5% (454)	1.023 (0.839–1.248)	0.82
Late level	42.8% (71)	0.881 (0.625–1.242)	0.47
Boys			
Early level	40.8% (151)	Reference	
Middle level	44.5% (241)	1.165 (0.891–1.523)	0.26
Late level	37.3% (31)	0.865 (0.529–1.412)	0.56
Girls			
Early level	52.4% (151)	Reference	
Middle level	48.9% (213)	0.867 (0.643–1.167)	0.35
Late level	48.2% (40)	0.844 (0.518–1.376)	0.50

CI = confidence interval.

## Discussion

To our knowledge, this study provides the first school-based longitudinal observational with cycloplegic data on the association between sleep duration, bedtime, and myopia progression over a 4-year follow-up period in Chinese primary school children. At the last follow-up, the mean myopia progression and axial elongation for all children were –1.89 ± 1.28 D and 1.22 ± 0.57 mm. In multiple linear regression models, there was no significant association between sleep duration and bedtime with myopia progression and axial elongation in primary school children. When the analysis was split by sex, the association between sleep duration and axial elongation in girls is only of borderline significance.

The current data on sleep duration were compared with sleep durations reported in previous studies. In our study, the mean sleep duration of children was 9.83 ± 0.58 h/d. Our result was similar to sleep duration reported in previous studies of Chinese children in different cities.^23^^,^^24^ For example, in a cross‑sectional study of 9198 Chinese children aged 3 to 14 years, Li et al.^23^ found that the daily sleep duration of children in Beijing was 9.7 ± 0.7 h/d. However, the current data on sleep duration were shorter than those in Spain, Australia, and the United States.^25^

Previous reports have shown an inverse association between myopia and sleep duration in children.^15^^,^^16^ After adjusting for potential confounders, Jee et al.^15^ found that the adjusted odds ratio for myopia was decreased in those with less than 9 hours of sleep in Korean adolescents aged 12 to 19 years (odds ratio, 0.59; 95% confidence interval, 0.38–0.93) than in those with less than 5 hours of sleep.^15^ Ayaki et al.^16^ also found that shorter sleep duration in children aged 10 to 19 years was significantly correlated with high myopia (*β* = −0.194; *P* = 0.001) in the simple regression analysis. However, our study showed that sleep duration was not significantly associated with SE and AL at baseline for all children. The noncycloplegic refraction and different ages as well as the lack of controlling for confounding variables in these studies, may contribute to the conflicting results. The current evidence has suggested that noncycloplegic refraction is thus problematic in epidemiologic studies, particularly in studies addressing questions of associations.^26^ In addition, these studies were not adjusted by key confounders, such as outdoor activity and near work in school children. Owing to early intense educational pressures in the school years in East Asia,^9^ students may spend less time in outdoor activities and more time in sustained near work before bedtime, leading to the development of a high prevalence of myopia. The amount of near work done before bedtime might be related to a shorter sleep duration. In addition, different types of near work activities the children might be doing before bedtime, such as doing homework, the exposure to display screen equipment, and playing activities, may have different effects.

The relationship of sleep duration with myopia progression and axial elongation has been seldom reported. The Growing Up in Singapore Towards Healthy Outcomes birth cohort examined 376 children and found that the sleep duration at 12 months of age was not associated with refractive error at 3 years.^18^ Another study recruiting 137 law students with a mean age of 27 years at the University of Pennsylvania also suggested that sleep duration did not correlate with myopia progression.^19^ Similarly, this relationship was not found in our study for all children. In addition, we found that the incidence of myopia was not associated with sleep duration in univariate logistic regression analysis. However, when the analysis was split by sex, we found that myopia progression and axial elongation over 4 years were inversely related to sleep duration in girls but not in boys after stratifying the sleep duration into tertile groups. In multiple linear regression models, the association between sleep duration and axial elongation in girls is only of borderline significance (*P* = 0.045). The magnitude of effects was clinically small. In previous study, Ayaki et al.^16^ found that bedtime was significantly associated with myopic refractive error in the regression analyses in Japanese children aged 10 to 19 years. However, this association was not found in our study. We suspect it was mostly caused by the noncycloplegic refraction performed in their study, because noncycloplegic refraction leads to errors in the estimation of SE and refractive categories. Additionally, the narrow bedtime range (88.7% of the children's bedtime fell between 8:30 and 10:00 PM in our study) may also partially explain the inconsistent results.

A related hypothesis for explaining these associations in girls is that the earlier onset of puberty in girls may occur concomitantly with the faster progression of myopia. Previous studies had demonstrated that girls grow at their fastest rate early in puberty, whereas boys grow at their fastest rate near or after the middle of puberty.^27^^,^^28^ Therefore, myopia progression and axial elongation are larger owing to differences in sleep duration among primary school girls. In our study, the respective mean myopia progression values for girls and boys were –2.10 ± 1.30 D and –1.71 ± 1.25 D during the 4-year follow-up in primary school children; girls demonstrated significantly higher myopic progression than boys (*P* < 0.001).

Several potential mechanisms have been proposed to explain the relationship between myopia and sleep duration. However, the exact mechanisms remain unclear at present. A traditional hypothesis has proposed that normal circadian rhythms are important to human eye development.^29^^,^^30^ It seems reasonable that these rhythms can be disrupted by shorter sleep duration and subsequently affect regulatory mechanisms controlling the emmetropization process of the eye. It was postulated that the biological pathways behind the association between sleep and myopia are related to retinal dopaminergic pathways. The fundamental regulator of sleep depends on the circadian cycle in melatonin synthesis and release controlling by reciprocal interactions with retinal dopaminergic pathways.^29^^,^^30^ A previous study showed that the downregulation of dopamine D2R in the ventral striatumin in humans, caused by sleep deprivation (evidence that sleep deprivation downregulates dopamine d2r in ventral striatum in the human brain),^31^ may lead to less activation of retinal dopaminergic pathways, resulting in axial elongation.^18^ It is also possible that high educational intensity can result in amounts of near work and less time in outdoor activities before bedtime among school children, leading to a shorter sleep duration. Studies have found that more time spent in near work and less time in outdoor activities seem to contribute to the pathogenesis of myopia.^12^^,^^22^ Such inverse associations between sleep duration and myopia could be confounded by more time in school-related near work, and sleep deprivation may be a covariate.

Strengths of the present study include the standardized measurement of refractive errors using cycloplegia, a relatively large sample size that adjusted for potential confounders, as well as the fact that this is the first cohort study investigating the effects of sleep duration on myopia progression and axial elongation in school children. However, our study also had limitations. The main weakness of our study was that sleep duration was obtained by parental report through questionnaires as well as other studies, which may be subject to recall bias. In addition, our study did not investigate sleep quality, even though some studies have shown that sleep quality may be related to myopia.

## Conclusions

There was no significant association between sleep duration and bedtime with myopia progression and axial elongation in primary school children. The findings in girls might be related to the earlier onset of puberty. Further studies should be conducted to clarify in detail the association between sleep duration and myopia progression and axial elongation in different age groups and ethnic populations.
